# Thoracic impedance measures tissue characteristics in the vicinity of the electrodes, not intervening lung water: implications for heart failure monitoring

**DOI:** 10.1007/s10877-014-9570-x

**Published:** 2014-03-12

**Authors:** Christopher J. Charles, Miriam T. Rademaker, Iain C. Melton, Dan Gutfinger, Neal L. Eigler, Fujian Qu, Richard W. Troughton

**Affiliations:** 1 Christchurch Heart Institute, University of Otago, P.O. Box 4345, Christchurch, New Zealand; 2 Implantable Electronic Systems Division, St. Jude Medical, Sylmar, CA USA; 3 Division of Cardiology, Cedars-Sinai Medical Center, UCLA School of Medicine, Los Angeles, CA USA

**Keywords:** Implantable monitors, Hemodynamics, Left atrial pressure

## Abstract

The rationale for intrathoracic impedance (Z) detection of worsening heart failure (HF) presupposes that changes in Z reflect changes in pulmonary congestion, but is confounded by poor specificity in clinical trials. We therefore tested the hypothesis that Z is primarily affected by tissue/water content in proximity to electrodes rather than by lung water distribution between electrodes through the use of a new computational model for deriving the near-field impedance contributions from the various electrodes. Six sheep were implanted with a left atrial pressure (LAP) monitor and a cardiac resynchronization therapy device which measured Z from six vectors comprising of five electrodes. The vector-based Z was modelled as the summation of the near-field impedances of the two electrodes forming the vector. During volume expansion an acute increase in LAP resulted in simultaneous reductions in the near-field impedances of the intra-cardiac electrodes, while the subcutaneous electrode showed several hours of lag (all *p* < 0.001). In contrast, during the simulated formation of device-pocket edema (induced by fluid injection) the near-field impedance of the subcutaneous electrode had an instantaneous response, while the intra-cardiac electrodes had a minimal inconsistent response. This study suggests that the primary contribution to the vector based Z is from the tissue/water in proximity to the individual electrodes. This novel finding may help explain the limited utility of Z for detecting worsening HF.

## Introduction

Despite treatment advances and more intensive clinical monitoring, heart failure (HF) remains one of the most common causes for hospitalization and is associated with high morbidity, mortality and economic costs [1]. The increasing use of implantable cardioverter defibrillators (ICD) and cardiac resynchronization therapy (CRT) devices in patients with HF makes it possible to remotely monitor intrathoracic impedance (Z) in combination with other physiological parameters [2]. Z measurements using the right ventricular (RV) shocking coil to the device case vector (RV_coil_-Case) are traditionally thought to represent the resistance to electrical flow across a field extending through the lungs, such that changes in Z can be interpreted to reflect changes in thoracic fluid volume and pulmonary congestion [3, 4]. Based on this premise several studies have shown that it is possible to use Z to predict and potentially reduce hospitalizations for acute decompensated HF (ADHF) [5, 6]. However, because of the limited specificity and sensitivity of Z monitoring when using the RV_coil_-Case vector [5, 7], more recent studies have explored the use of alternative and combination vectors from CRT leads [8–10].

Pre-clinical studies using multiple vectors have shown that each vector changes at a different rate and magnitude in response to the development of pacing-induced HF [9, 10]. The left ventricular (LV) ring electrode to device case vector (LV_ring_-Case) demonstrated the fastest rate and greatest magnitude of change, and correlated well with left atrial pressure (LAP). A recent clinical study reported that a combination of Z vectors, rather than Z from a single vector can successfully identify increases in thoracic fluid volume that will culminate in ADHF. Using a combination vector from either CRT or ICD leads showed improved sensitivity and a lower false positive rate compared with the single RV_coil_-Case vector [11].

In order to more effectively exploit the availability of Z from multiple vectors, we sought to gain better insight into what is being measured by these vectors. The electric current flowing between two electrodes forming a vector does not necessarily travel along a straight line across the lungs, but more likely along pathways of lesser resistance. We, therefore, hypothesized that the most significant contribution to the measured Z is from the electrode-tissue interface where there is high impedance to electrical flow, and that beyond this interface the contribution to the measured Z is negligible [12]. This is because beyond the electrode-tissue interface the current finds its way to flow through low resistance structures, such as blood vessels, that do not have a significant contribution to the measured Z. If Z primarily depends on the electrode-tissue interface, then it provides a measure of the local tissue characteristics surrounding the two electrodes rather than the characteristics of tissues located farther away between the electrodes. This new interpretation may help explain why recent clinical trials have shown that monitoring of Z has limited effectiveness in detecting ADHF [13, 14], particularly in the early months post-device implant when there are significant non-HF related changes occurring at the electrode-tissue interface. To test the hypothesis that Z primarily reflects local tissue characteristics we evaluated a new computational model for deriving the near-field impedance contributions from the various electrodes and conducted a pre-clinical experiment to illustrate how near-field impedances may be monitored at multiple sites.

## Methods

The study protocol was approved by the Animal Ethics Committee of the University of Otago, Christchurch, New Zealand.

### Implantation Procedure and Devices

Under general anesthesia (induced by 15 mg/kg thiopentone; maintained by halothane/nitrous oxide inhalation) six sheep were implanted via the right jugular vein with an LAP monitor (HeartPOD™ ISL) and three standard transvenous bipolar pacing/defibrillation leads with the tip electrodes fixed in the right atrial (RA) appendage, RV apex and LV epicardial region via the coronary sinus. The leads were attached to a CRT device (Promote™ RF) that was implanted subcutaneously over the left chest wall. The CRT device provided Z measurements along six vectors: V1 = LV_ring_-Case; V2 = RV_ring_-Case; V3 = RA_ring_-Case; V4 = RV_coil_-Case; V5 = LV_ring_-RV_ring_; and V6 = LV_ring_-RA_ring_ [9, 10]. The LAP monitor was implanted into the left atrium via a trans-septal catheterization, and provided direct LAP waveform measurements via telemetry communication [15, 16]. All implanted devices were manufactured by St Jude Medical, Implantable Electronic Systems Division in Sylmar, CA, USA. Post-operatively, animals recovered for a minimum of 5 weeks to allow the Z measurements to stabilize.

### Acute study protocols

Following the stabilization period, sheep were studied on separate days as follows:

#### Intravascular volume expansion in normal sheep (pre-pacing)

Sheep received a 4-h intravenous (IV) infusion of Dextran (initial dose 15 ml/kg over 30 min, then titrated to maintain LAP at approximately 15 mmHg above baseline values).

#### Induction of Congestive HF

Sheep underwent 7 days of rapid RV pacing at 190 bpm to induce a state of moderate stable congestive HF. This rate of RV pacing consistently raised LAP levels in this animal model by approximately 10 mmHg [17].

#### Intravascular volume expansion during congestive HF

Sheep received a 4-h IV infusion of Dextran (as above) in the HF state. Dextran was titrated to raise LAP by approximately 15 mmHg (from HF baseline levels of 16–17 mmHg to approximately 31–32 mmHg).

#### Device pocket edema

To simulate the formation of edema within the device pocket 250 mL of 0.9 % saline was rapidly injected via an angiocath catheter into the subcutaneous pocket containing the CRT device case. An additional 250 mL saline bolus was injected in 5/6 sheep 2 h later.

### Data Acquisition and Processing

**Fig. 1:**
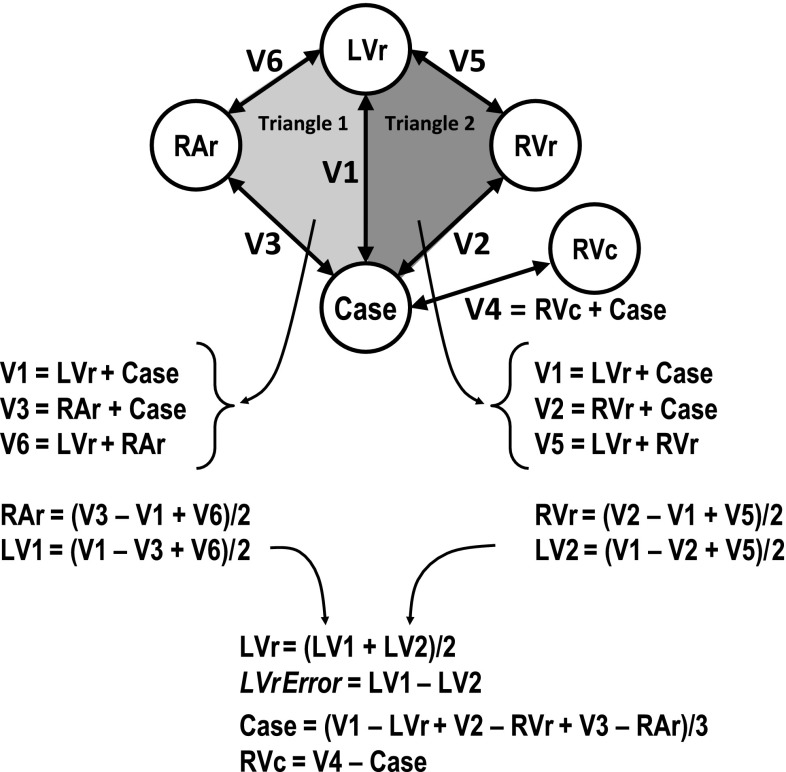
Methodology for calculating near-field impedance by solving the algebraic equations of the vector-based (V1–V6) impedance measurements

LAP measurements were recorded every 5 min throughout the acute study protocols as previously described [15, 16]. Z measurements were automatically collected through the CRT device along all six vectors (V1 through V6) at 7.5 min intervals throughout the acute study protocols. Using equivalent circuit equations [18], the measured Z along each vector was modelled as the sum of the near-field contributions from the two measuring electrodes to produce a set of six linear equations (Fig. 1). The set of equations was solved algebraically to determine the near-field impedance contribution (denoted by Z_E_ or the electrode name) associated with each of the five electrodes. As outlined in Fig. 1, the Z_E_ associated with the LV_ring_ electrode was computed using the equations corresponding to two different impedance triangles to yield LV1 and LV2 which were then averaged. The difference between LV1 and LV2 determined the measurement error associated with the computational model.

### Statistics

All results are expressed as mean ± SEM unless otherwise specified. For each acute study the effect on LAP, Z and Z_E_ parameters were assessed using one-way repeated measures analysis of variance (ANOVA). A value of *p* < 0.05 was considered statistically significant.

## Results

### Near-field impedance measurement validation

Near-field impedances (Z_E_) and the relative contributions from each of the five electrodes toward the vector-based Z measurements are summarized in Table 1. As expected, the highest Z_E_ was associated with the smaller sized electrodes (LV_ring_, RV_ring_ and RA_ring_), while the lowest Z_E_ was associated with the larger sized electrodes (Case and RV_coil_). Electrode size also influenced the relative contributions from each of the electrodes towards the measured Z. For a vector between any ring electrode (LV_ring_, RV_ring_, and RA_ring_) and the Case electrode (V1 through V3), most of the contribution to Z was found to be from the ring electrode (77-87 %), while only 13–23 % of the contribution was from the Case. In contrast, for the RV_coil_ to Case vector (V4) most of the contribution to Z was found to be from the subcutaneous Case electrode (69 %), with only 31 % from the intra-cardiac RV_coil_ electrode. For the intra-cardiac vectors that are between two ring electrodes (V5 and V6) the contributions from the two electrodes was found to be similar. Thus, it appears that for a vector with different electrode sizes, the smaller electrode or the electrode that is surrounded by more scar tissue will be the one that is most influencing any changes in the vector-based Z measurement.

**Table 1:** Mean impedance measurements with relative contribution of near-field Z_E_ to vector-based Z. Data averaged across all sheep (N = 6) for all measurements over entire duration of implants [100 ± 22 (SD) days]

Vector	Mean ± SD Z (Ω)	Contribution from 1st electrode	Contribution from 2nd electrode	Electrode	Mean ± SD Z_E_ (Ω)
V1: LV_ring_-Case	306 ± 53	87 % LV_ring_	13 % Case	LV_ring_	266 ± 50
V2: RV_ring_-Case	177 ± 29	77 % RV_ring_	23 % Case	RV_ring_	137 ± 26
V3: RA_ring_-Case	254 ± 28	84 % RA_ring_	16 % Case	RA_ring_	214 ± 26
V4: RV_coil_-Case	58 ± 6	31 % RV_coil_	69 % Case	Case	40 ± 5
V5: LV_ring_-RV_ring_	403 ± 50	66 % LV_ring_	34 % RV_ring_	RV_coil_	18 ± 5
V6: LV_ring_-RA_ring_	480 ± 58	55 % LV_ring_	45 % RA_ring_		
				LV1	268 ± 50
				LV2	265 ± 50

The error in computing the near-field impedance for the LV_ring_ electrode (Fig. 1) was found to be extremely small at 2.9 ± 3.8 Ω (~1 %). This small error in combination with the observations regarding electrode size influencing Z_E_ both validate the computational model used for deriving the near-field impedance contributions to the vector-based Z measurements.

### Intravascular volume expansion in normal sheep (pre-pacing)

**Fig. 2:**
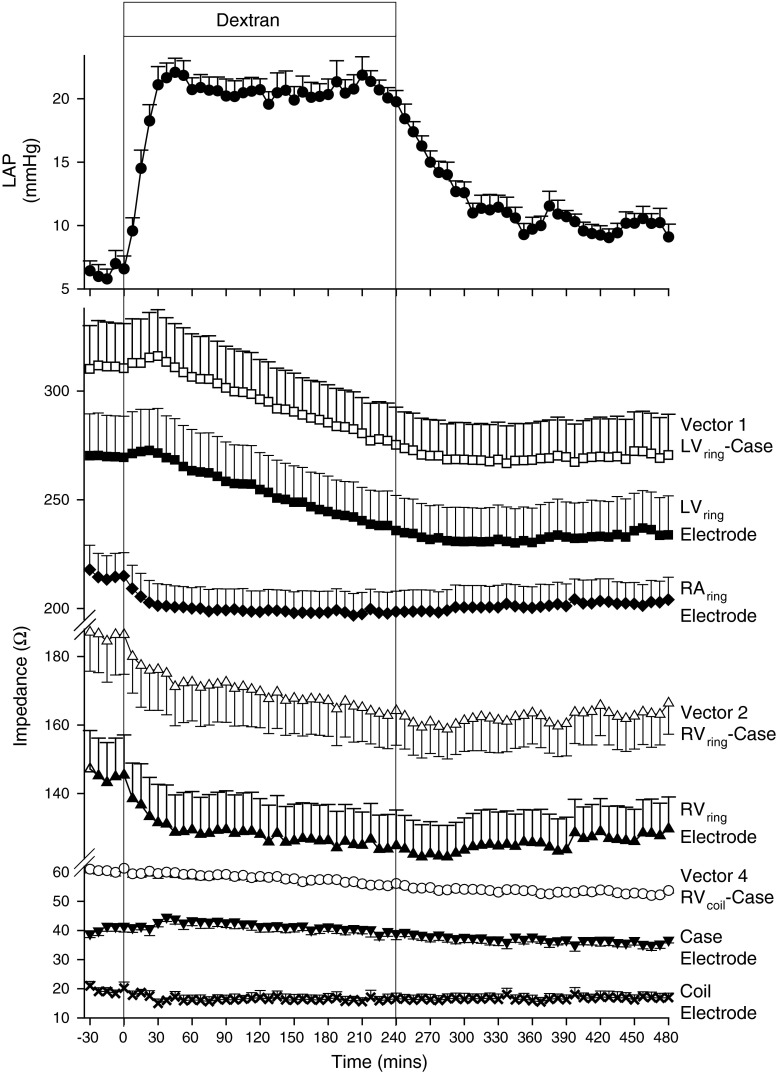
Mean ± SEM LAP, vector-based and near-field impedance responses to acute intravascular volume expansion in six normal sheep

The total volume of Dextran administered over the 4 h infusion period ranged between 3,500 and 4,500 mL. LAP increased from 7 ± 1 to 21 ± 1.4 mmHg by 30 min and remained stable while Dextran was being infused (Fig. 2). Once the infusion terminated, LAP gradually fell over 60 min, but remained 2–3 mmHg above baseline levels for the remainder of the recording period. In comparison, after a brief delay (30 min), LV_ring_-Case Z fell (*p* < 0.001), reaching a nadir at 285 min and remaining low. Z_E_ from LV_ring_ (*p* < 0.001) showed a similar response, achieving a nadir after 270 min. RV_ring_-Case Z fell without delay reaching a nadir at 265 min (*p* < 0.001). In contrast, RV_coil_-Case Z demonstrated a delayed fall (90 min), and did not reach nadir till 375 min (*p* < 0.001). The RV_ring_, RV_coil_ and RA_ring_ Z_E_ all fell immediately and abruptly (all *p* < 0.001) mirroring the acute change in LAP, and reaching nadirs at 45, 30 and 30 min, respectively. Despite the observation that LAP fell within 60 min following Dextran infusion, the intra-cardiac Z_E_ measurements remained suppressed for the duration of the recording period. Case Z_E_ changes were less consistent, with the level actually rising initially and ultimately reducing with significant lag.

### Induction of congestive HF

Pacing at 190 bpm induced a moderate state of HF as indicated by rapid and significant increases in LAP, rising from a pre-pacing baseline of 7 ± 1–16 ± 1 mmHg within a few hours of pacing, before plateauing (*p* < 0.001). Pacing induced the expected decreases in vector-based Z, falling from pre-pacing levels of 310 ± 21–257 ± 21 Ω, 186 ± 12–162 ± 10 Ω and 61 ± 1–54 ± 2 Ω for LV_ring_-Case, RV_ring_-Case and RV_coil_-Case, respectively (all *p* < 0.001). Similarly, pacing produced falls in the Z_E_ for LV_ring_ (269 ± 19–220 ± 19 Ω, p < 0.001), RV_ring_ (145 ± 12–125 ± 19 Ω, *p* = 0.004) and Case (41 ± 2.1 vs. 37 ± 1.9 Ω, *p* = 0.014) electrodes. The Z_E_ of the RV_coil_ (20.2 ± 2.1–17.5 ± 2.1 Ω, *p* = 0.07) and RA_ring_ (215 ± 11–203 ± 11 Ω, *p* = 0.16) electrodes also tended to decline.

### Intravascular volume expansion during congestive HF

**Fig. 3:**
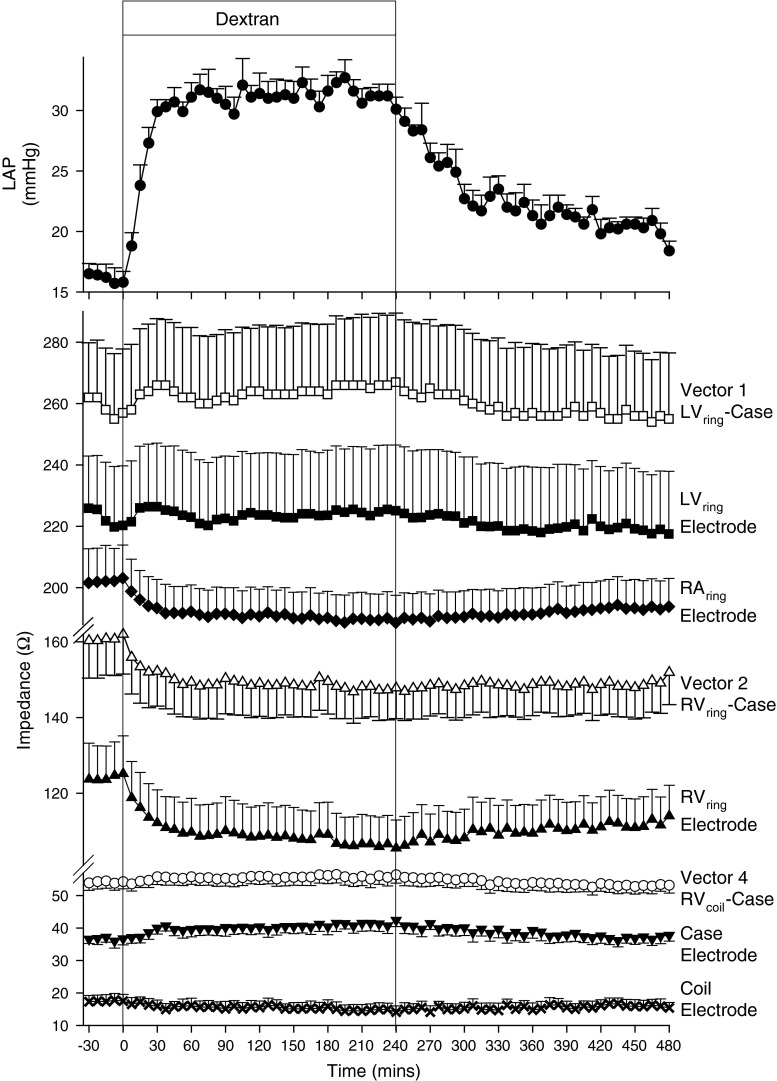
Mean ± SEM left atrial pressure (LAP), vector-based and electrode-based impedance responses to intravascular volume expansion with Dextran in six sheep with stable pacing-induced HF

The total volume of Dextran administered over the infusion period was 2,900–4,000 mL. Acute volume loading increased LAP from 16 ± 1 to 31 ± 1.2 mmHg by 30 min, with pressures remaining stable for the duration of the infusion (Fig. 3). LAP gradually reduced following termination of Dextran infusion, but remained above pre-Dextran infusion levels. Both LV_ring_-Case and RV_coil_-Case vectors showed a small but consistent rise (both *p* < 0.001) in response to Dextran (Fig. 3), with similar rises observed for Z_E_ of the LV_ring_ (*p* < 0.001) and Case (*p* < 0.001). In contrast, RV_ring_-Case vector-based Z and Z_E_ from RV_ring_, RA_ring_ and RV_coil_ all showed prompt and consistent falls (all *p* < 0.001), reaching nadirs by 37.5 min and remaining depressed for the duration of the recording period.

### Device pocket edema

**Fig. 4:**
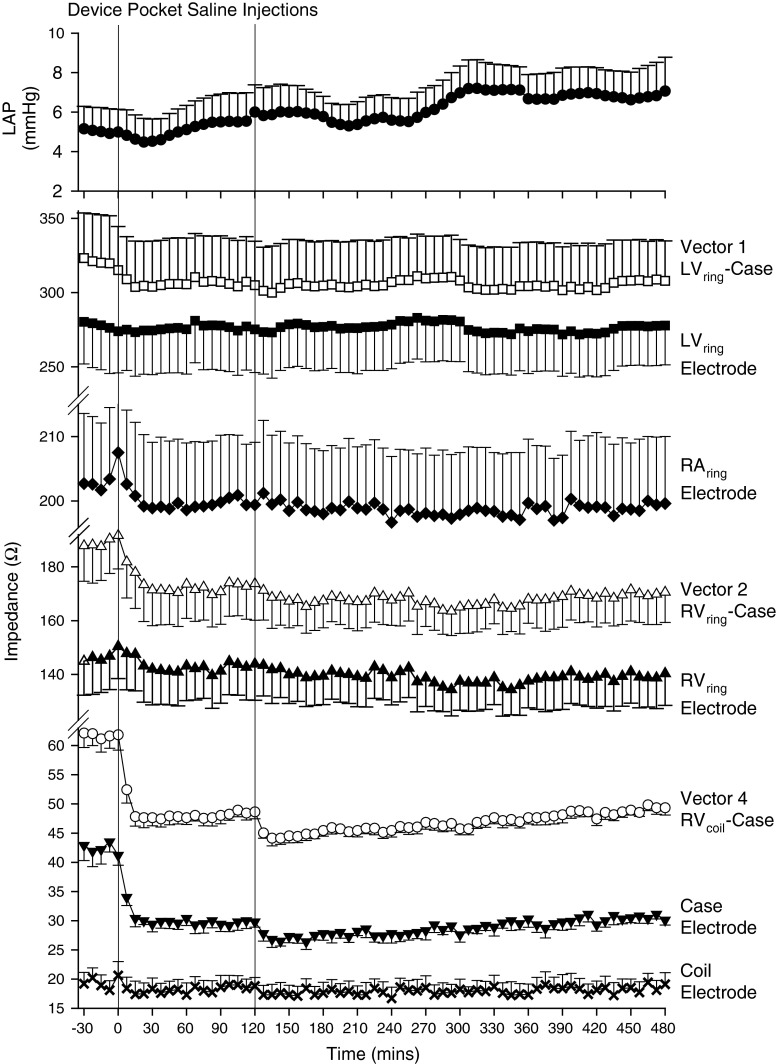
Mean ± SEM LAP, vector-based and near-field impedance responses to injection of fluid into the CRT-D device pocket in six normal sheep

There was a small but consistent elevation in LAP (~2 mmHg; *p* < 0.001) during the study day which appeared unrelated to fluid injection (Fig. 4). All three vector-based Z showed an immediate decline (all *p* < 0.001) in response to fluid injection into the device pocket, and remained lower than baseline. Z_E_ from the Case showed a prompt and dramatic fall in response to pocket fluid injection (*p* < 0.001), dropping from ~43 to ~30 Ω following the first injection, and then to ~27 Ω following the second injection. In contrast, Z_E_ associated with the other four electrodes showed minimal and inconsistent changes unrelated to fluid injection.

## Discussion

In this study we demonstrate for the first time that changes in vector-based Z measurements may readily develop in response to changes occurring at the electrode-tissue interface, thereby providing a possible mechanism that explains why Z monitoring has limited sensitivity and specificity in detecting ADHF [13]. The hypothesis that Z primarily reflects local tissue characteristics suggests that non-HF events, such as healing post-implant, lead migration/dislodgement, and device-pocket infection, can easily confound the interpretation of changes in Z measurements [4, 14]. Thus, in the current study we evaluated changes in both vector-based Z and Z_E_ responses during rapid changes in LAP induced by volume and non-volume-dependent mechanisms. Volume loading with dextran is a standard physiological model designed to acutely increase intravascular and intracardiac volumes [19, 20]. The rate and duration of dextran infusion in the current study was sufficient to increase intravascular and intracardiac volumes with an approximate 15 mmHg rise in LAP, but is unlikely to have caused pulmonary edema. Rapid ventricular pacing is a well established animal model of congestive HF which consistently induces significant sodium and water retention, changes in intracardiac volumes and pressures and pulmonary edema [17]. This model has been previously used in our lab to study vector-based impedance changes with the onset and offset of congestive HF, thereby modelling ADHF decompensation and recovery [10]. Results from the current study confirm that pacing induced HF not only reduces all vector-based impedances, as previously shown [10], but also consistently reduces Z_E_ derived from each of the electrodes.

Results from the current study support our hypothesis that Z primarily reflects local tissue characteristics by demonstrating that intrathoracic impedance measurements reflect the local tissue and fluid conditions surrounding each of the electrodes, rather than the characteristics of tissues located farther away in the region between the measuring electrodes. The highest near-field impedance (Z_E_) was associated with the smaller-sized LV_ring_, RV_ring_ and RA_ring_ electrodes which had greater resistance to electrical flow at the electrode-tissue interface (Table 1). The lowest Z_E_ was associated with the Case and RV_coil_ electrodes, consistent with these electrodes being larger in size with less resistance to electrical flow. The LV_ring_ electrode had the highest Z_E_, consistent with this electrode being surrounded by more tissue and less blood.

To construct the mathematical model we hypothesized that the current flows along a pathway from the first electrode to the second electrode which includes three components; (1) the tissues adjacent to the first electrode; (2) the tissues and/or blood located farther away between the two electrodes; and (3) the tissues adjacent to the second electrode. The measured impedance between the first electrode and the second electrode therefore can be modelled as the summation of the impedance associated with each of these pathway components. We also hypothesized that the contribution to the measured impedance between the first and the second electrode is greatest from the 1st and 3rd components of the pathway, and that the contribution from the 2nd component is significantly lower such that it may be ignored. We believe this hypothesis to be correct because the electrical current will find its way to flow through a pathway between the electrodes that has the lowest impedance to electrical flow, such as along blood vessels. Thus, the mathematical model for the measured impedance between two electrodes was simplified to consist of only the 1st and 3rd components. Since we had six measurements of vector based impedance from five electrodes we were able to compute the contribution from the LV_ring_ electrode in more than one way (LV1 and LV2) which allowed us to verify our hypothesis that the impedance associated with the 2nd component pathway can be ignored.

**Fig. 5:**
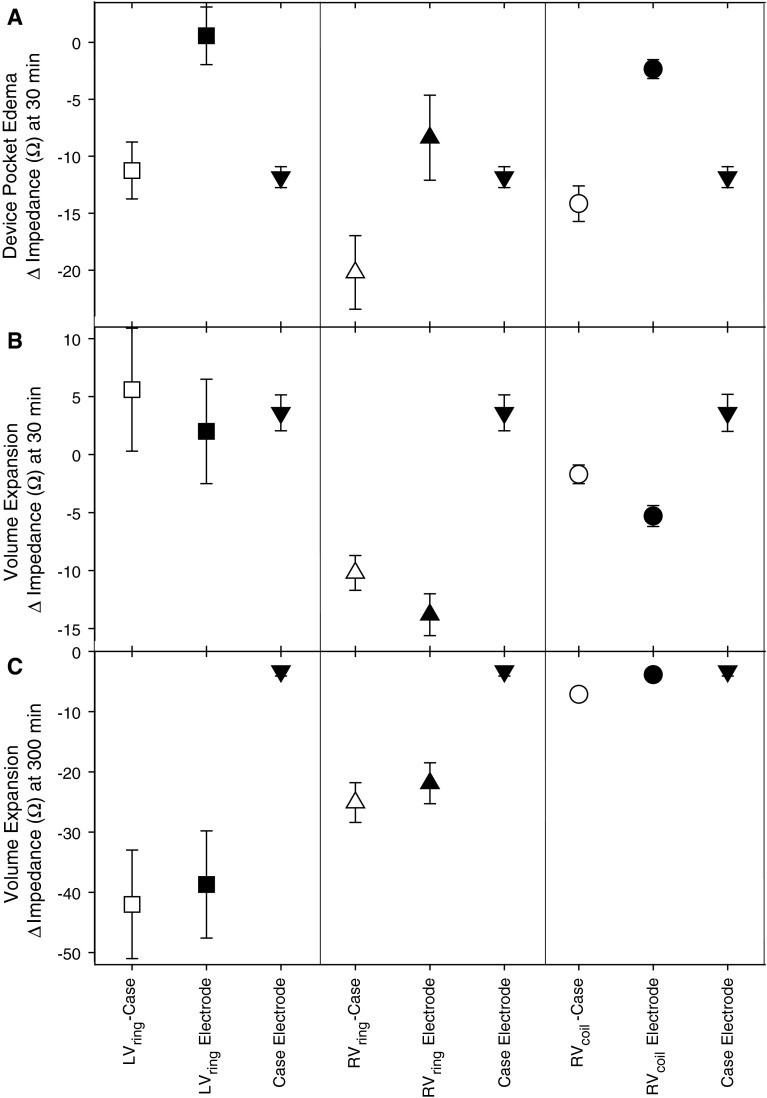
Relative contributions of near-field impedances to changes in vector based impedance measured in response to device pocket saline injection at 30 min (**a**); and intravascular volume expansion at 30 min (**b**) and 300 min (**c**)

The most striking example of impedance reflecting the local tissue and fluid conditions surrounding an electrode was seen in the device pocket edema study where, although vector-based Z (V1, V2, and V4) fell in response to the injection of fluid into the device pocket, near-field computation demonstrated that the marked and precipitous fall in the Case Z_E_ contributed most, if not all, to the decrease in the vector-based Z. This can be seen in Fig. 5a where the falls in Z for V1, V2, and V4 are plotted at 30 min following saline injection into the pocket. The reduction in Z for V1 and V4 is contributed almost exclusively by the fall in Z_E_ of the Case electrode, with little if any contribution from the Z_E_ of the LV_ring_ or RV_coil_ electrodes, respectively. The large accumulation of fluid surrounding the Case resulted in little discernable change in LAP or Z_E_ for any of the other implanted electrodes located far away from the device pocket. A similar response pattern in the measured Z is classically seen whenever a pulse generator is replaced, which results in the formation of local edema within the device pocket during the first 24 h, followed by the normal phases of tissue healing occurring over the subsequent weeks to months. Thus, device pocket edema induces significant reductions in the measured Z that may be falsely interpreted [4]. However, with the ability to remove the contribution of the Case electrode from the measured vector-based Z, it is possible to monitor impedance without it being subjected to otherwise confounding changes that are occurring within the device pocket.

The hypothesis that Z primarily reflects local tissue characteristics is further supported by the intravascular volume expansion studies performed both in normal (pre-pacing) and HF sheep which induced a significant increase in LAP that was promptly tracked by corresponding reductions in the Z_E_ measurements from the intra-cardiac (RV_ring_, RA_ring_ and RV_coil_) electrodes. The acute and the delayed responses to intravascular volume expansion in the normal (pre-pacing) sheep are illustrated in Fig. 5b and c, respectively. At 30 min following commencement of volume expansion, which corresponds to the time when LAP first peaked, there is a dramatic fall in Z for V2 and V4, whereas for V1 there is a slight unexpected increase (Fig. 5b). Near-field computation demonstrates that the acute reduction in Z for V2 and V4 is contributed entirely by a fall in the Z_E_ for the RV_ring_ and RV_coil_ electrodes, respectively. Because the RV_ring_ and RV_coil_ electrodes are located within the RV chamber, a rapid increase in chamber volume causes more fluid to surround the electrodes and less tissue to be in contact with the electrodes, such that the near-field contribution to the measured vector-based Z is proportionally reduced.

Following the 30 min time point the Z for V1 has a steady decrease (Fig. 2), which is occurring over the time period when the infused fluid is being redistributed to other compartments. At the 300 min time point (Fig. 5c) near-field computation demonstrates that virtually all of the reduction in Z for V1 is contributed by the fall in the Z_E_ for the LV_ring_ electrode. Although the LV_ring_ electrode is implanted within an epicardial blood vessel, its delayed response suggests that the LV_ring_ electrode is not in direct communication with the RV blood pool. This may be secondary to possible formation of scar tissue within the distal coronary venous implant site (confirmed by autopsy in some animals) causing obstruction to blood flow. Importantly, Z_E_ from the LV_ring_ electrode was found to be sensitive to congestion within the adjacent epicardial fat, as demonstrated post volume expansion in canine studies in our lab (unpublished observations), and to pericardial fluid volume and the degree of contact between the LV_ring_ electrode and the pericardial sac [21]. Thus, an increase in the degree of tissue contact between the LV_ring_ electrode and the pericardial sac as a consequence of an expanding cardiac chamber volume may explain the unexpected initial increase observed in response to acute volume infusion, while the later development of epicardial fat edema may explain the delayed response. A change in the degree of contact between the LV_ring_ electrode and the pericardial sac may also occur in response to changes in posture or pericardial fluid volume irrespective of pulmonary congestion, and may result in false interpretations of the measured impedance as previously reported [21]. However, the influence of free flowing pericardial fluid volume and posture may be less critical in patients with a scarred pericardial space following open heart surgery, or an episode of pericarditis.

There were variations in the recovery pattern of the measured Z_E_ following cessation of volume loading that depended on the electrode implant location. All five electrodes had a much slower recovery rate in Z_E_ (>12 h) compared to LAP (~3 h). The intra-cardiac right-sided electrodes demonstrated a faster recovery compared to the LV_ring_ and Case electrodes. The delay in recovery of impedance in comparison to LAP is consistent with previous reported findings [10], and is likely a consequence of the longer time required to restore the local fluid volume conditions surrounding the various electrodes, particularly when located outside the intravascular space. Similarly, termination of rapid ventricular pacing caused a precipitous acute fall in LAP, followed by a gradual recovery over the subsequent 12 h. This was in contrast to the significantly slower recovery seen in Z_E_ (~3 days—data not shown), which is consistent with previously reported data for Z_V_ [10]. The lag seen between Z_E_ and LAP is likely a consequence of Z_E_ being more a reflection of the local volume and corresponding tissue contact rather than pressure.

### Illustrative case studies

1. *Micro*-*dislodgement*. Figure 6 provides an example of the Z measurements acquired from a canine surgically prepared in a similar manner to the sheep in this study [9]. This animal developed a micro-dislodgement of the LV lead during the third week post-implant. The trend data demonstrates that during the first 24 h post-implant there is a significant decrease in the measured Z across all six vectors, which is consistent with the development of edema in the vicinity of all five electrodes. During the subsequent weeks the Z measurements gradually increased as the local edema resolved. However, there is a sudden disturbance in the measured Z on day 18 post-implant seen across the three vectors having the LV_ring_ in common (V1, V5, and V6). Examination of the Z_E_ shows that only the LV_ring_ electrode recorded a significant increase, with no change noted in the contribution from the other four electrodes. The ability to isolate the micro-dislodgement event into a single electrode additionally supports the computational model used to derive the near-field impedance contributions.

**Fig. 6:**
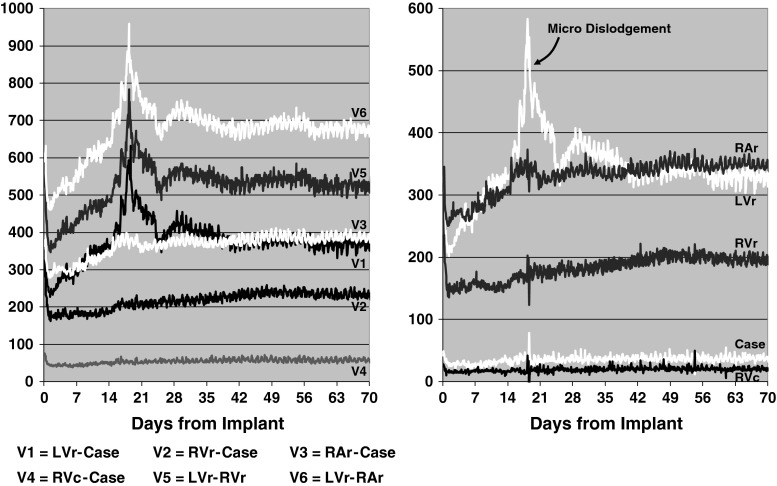
Example of vector-based (*left panel*) and near-field (*right panel*) impedance trend plots from a canine implanted with pacing/defibrillation leads using identical methodology to that described for sheep in this study. A micro-dislodgement of the LV lead occurred on day 18
2. *Hospitalization for ADHF*. An example of Z_E_ data acquired from a 66 year-old patient with ischemic cardiomyopathy is shown as a trend plot over time in Fig. 7. This patient provided informed consent to participate in the clinical trial reported by Binkley and colleagues [11]. Approximately 2 months following implant, the patient developed an episode of persistent atrial fibrillation that resulted in hospitalization for ADHF. During hospitalization, the patient was treated with intravenous diuretic therapy, and underwent an AV-node ablation procedure (Fig. 7). In response to this treatment, the Z_E_ from the RV_ring_ and Case electrodes increased dramatically, while the rise for the LV_ring_ Z_E_ was more gradual and continued post-hospital discharge. Interestingly, the Z_E_ from the RV_coil_ and RA_ring_ electrodes had more noise and measurement artifact, and did not coincide with the clinical course. This case illustrates that the response seen in Z_E_ during the development and recovery from ADHF varies with the electrode location and the local tissue characteristics.

**Fig. 7:**
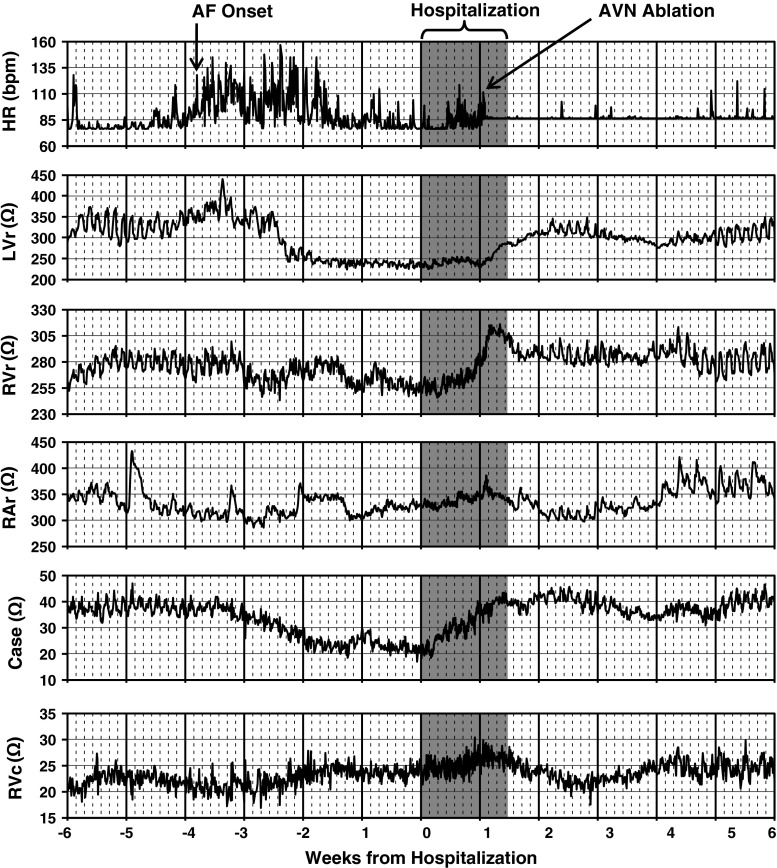
Example of near-field impedance trend plots shown from a patient with ischemic cardiomyopathy. Patient was hospitalized following an episode of persistent atrial fibrillation (AF). During hospitalization patient received both intravenous diuretic therapy and AV node ablation (AVN)

## Summary

Taken together, the results of the present study validate the computational model used to derive the near-field impedances (Z_E_), and demonstrate that vector-based Z primarily depends on the impedance from the tissues in the vicinity of the measuring electrodes, and therefore, reflects local tissue characteristics. This finding may explain why vector-based Z can display changes that do not coincide with pulmonary congestion, particularly in the early months post-implant when there are significant changes occurring at the electrode-tissue interface. This is consistent with the results from a recent clinical trial which demonstrate that the positive predictive value of a Z monitoring algorithm is particularly low in the initial 6 months post-implant, but subsequently improves over time [13]. Instabilities post-implant are more likely to occur with Z that are associated with a passive fixation lead, such as an LV pacing lead, where there is a greater chance for a micro-dislodgement to occur (Fig. 6). Therefore, it may be preferable to allow more time for the electrode-tissue interface to stabilize before relying on Z measurements for clinical decision making, or to rely on Z vectors that are derived from an active fixation lead (RV_coil_-Case and RV_ring_-Case). Once the electrode-tissue interface is stabilized, this study demonstrates that the Z_E_ associated with the intra-cardiac electrodes (RV_ring_, RA_ring_, and RV_coil_) respond faster than the Z_E_ associated with the LV_ring_ and Case electrode to an expanding intra-cardiac chamber volume. The Z_E_ associated with the LV_ring_ and Case electrodes are additionally influenced by changes occurring within the pericardial space or device pocket, which may not coincide with pulmonary congestion. Therefore, relying on the Z_E_ associated with the intra-cardiac electrodes for clinical decision-making may be more advantageous than other electrodes or Z vectors.

## Conclusion

We have reported for the first time a validated method for determining the relative contributions to the measured vector-based impedance from the various electrodes, and have demonstrated that vector-based impedance measurements predominantly reflect physical phenomena occurring in the vicinity of the measuring electrodes rather than farther away within the pulmonary parenchyma. Thus, the near-field computational model offers a new paradigm for examining impedance data from multi-lead devices which may prove clinically-useful in monitoring changes at specific anatomical locations in close proximity to the individual recording electrodes. The results of this study help explain why vector-based impedance measurements may display changes that do not necessarily correlate with the presence of pulmonary congestion, and support the hypothesis that intrathoracic impedance actually measures local tissue characteristics rather than intervening lung water.
